# Supplementary material for: Label-Free Imaging of Matrix Mineralization in Alginate-Encapsulated Bone Spheroids Using Coherent Raman Scattering Microscopy
Source: Chem Biomed Imaging. 2026 Mar 23;4(8):1837–49. doi: 10.1021/cbmi.6c00026 (PMC13508476; doi:10.1021/cbmi.6c00026)
Supplement: Supplementary file 1 [file im6c00026_si_001.pdf]

Supporting Information

Label-free imaging of matrix mineralization in alginate-encapsulated bone spheroids using Coherent Raman Scattering microscopy

Diamante Boscaro*[1], Sebastian J Kjeldgaard-Nintemann[2], Astrid Bjørkøy[1], and Pawel Sikorski[1]

[1]Department of Physics, Faculty of Natural Sciences, Norwegian University of Science and Technology (NTNU), Trondheim, 7491, Norway
[2]Center for Advanced Bioimaging, Department of Plant and Environmental Sciences, University of Copenhagen, Copenhagen, 1871, Denmark

*Email: diamante.boscaro@ntnu.no

Supplementary Section 1 Material and methods

Raman spectroscopy

Raman spectra were acquired on a Witec Alpha 300r Raman microscope. A 532 nm laser (100 mW) with a 600 grooves/mm grating was used. The spectra were collected using a 10x/0.25 objective. The integration time was set to 50 seconds per accumulation, with 6 accumulations used to create the spectra and laser power set to 10 mW. The samples, prepared and fixed following the same procedure as for ARS, prior to the analysis were peeled off the plastic well plate and moved to a calcium fluoride slide to avoid background from the substrate. The spectra were normalized using the asymmetric least squares to remove background fluorescence originating from the sample.

Supplementary Section 2 Results and Discussion

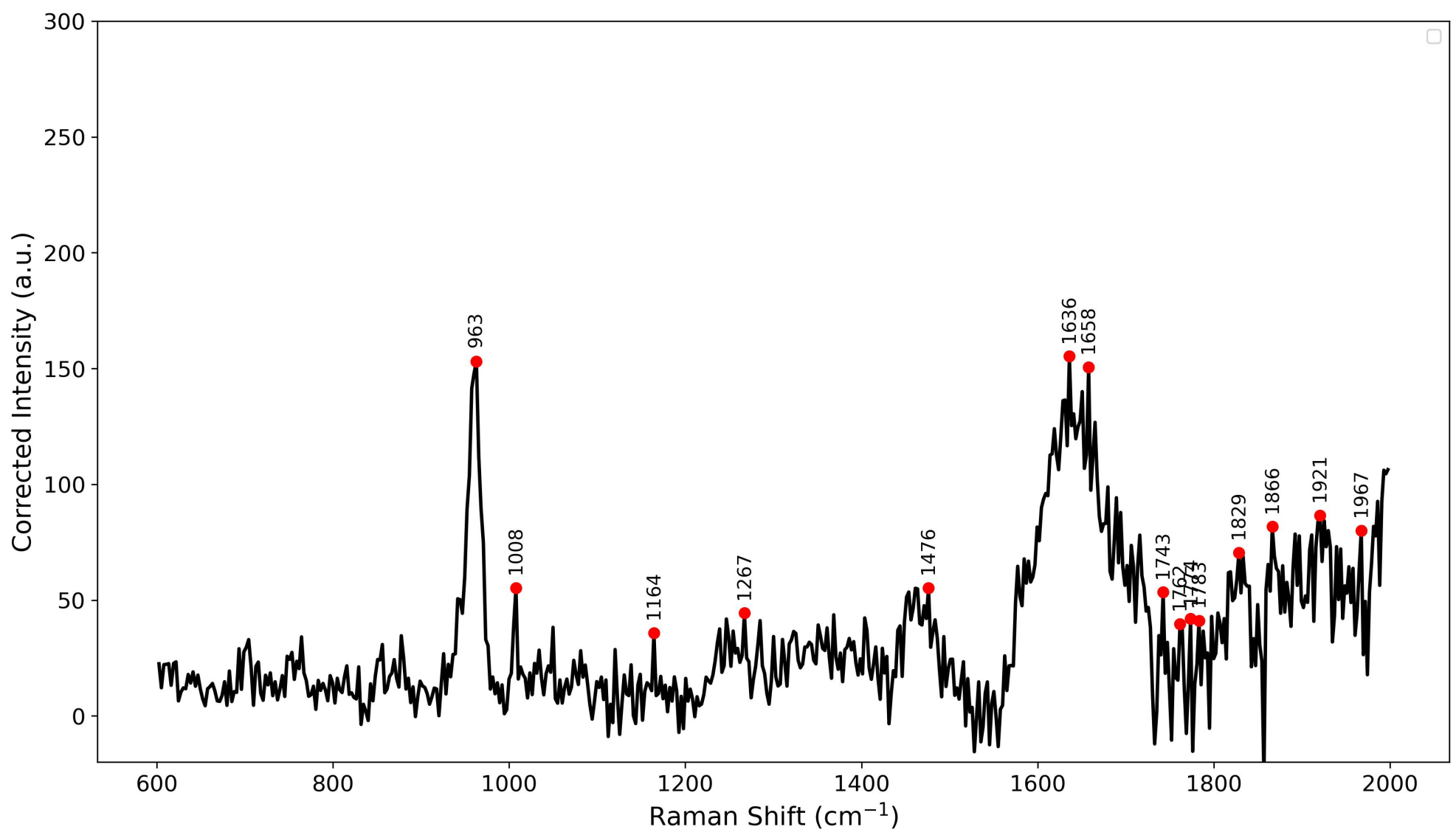

Figure S1: Spontaneous Raman spectra obtained from MC3T3-E1 monolayer cells cultured in OM for 3 weeks. The normalized spectra shows a dominant peak at 963 cm^{-1}, corresponding to the phosphate group, and smaller peaks at 1164, 1267, 1636 and 1658 cm^{-1} associated with deposited collagen.

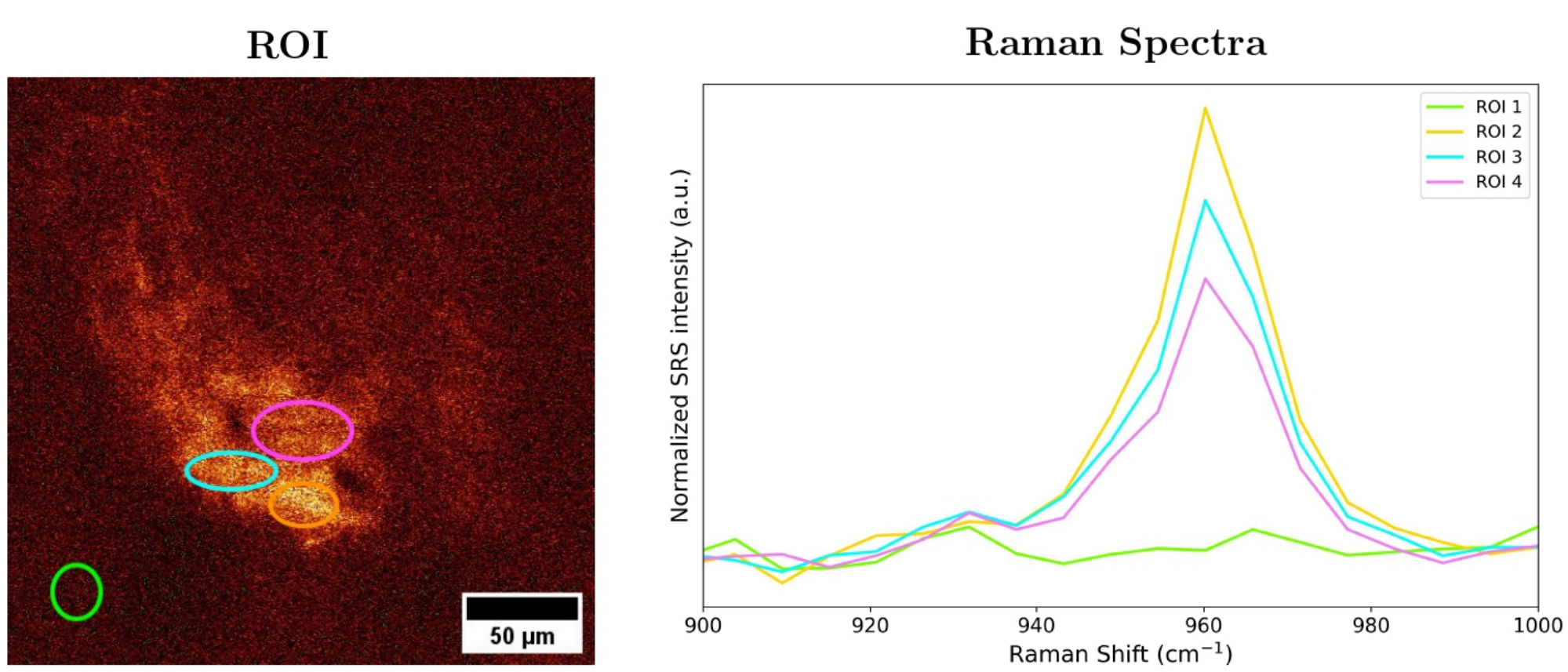

Figure S2: Raman spectra acquired in the 900 to 1000 cm^{-1} range and corresponding SRS image, indicating the selected regions of interest (ROIs).A distinct peak at 960 cm^{-1} is observed for ROIs centered in SRS-positive regions, while no peak is detected for the ROI selected from the background.

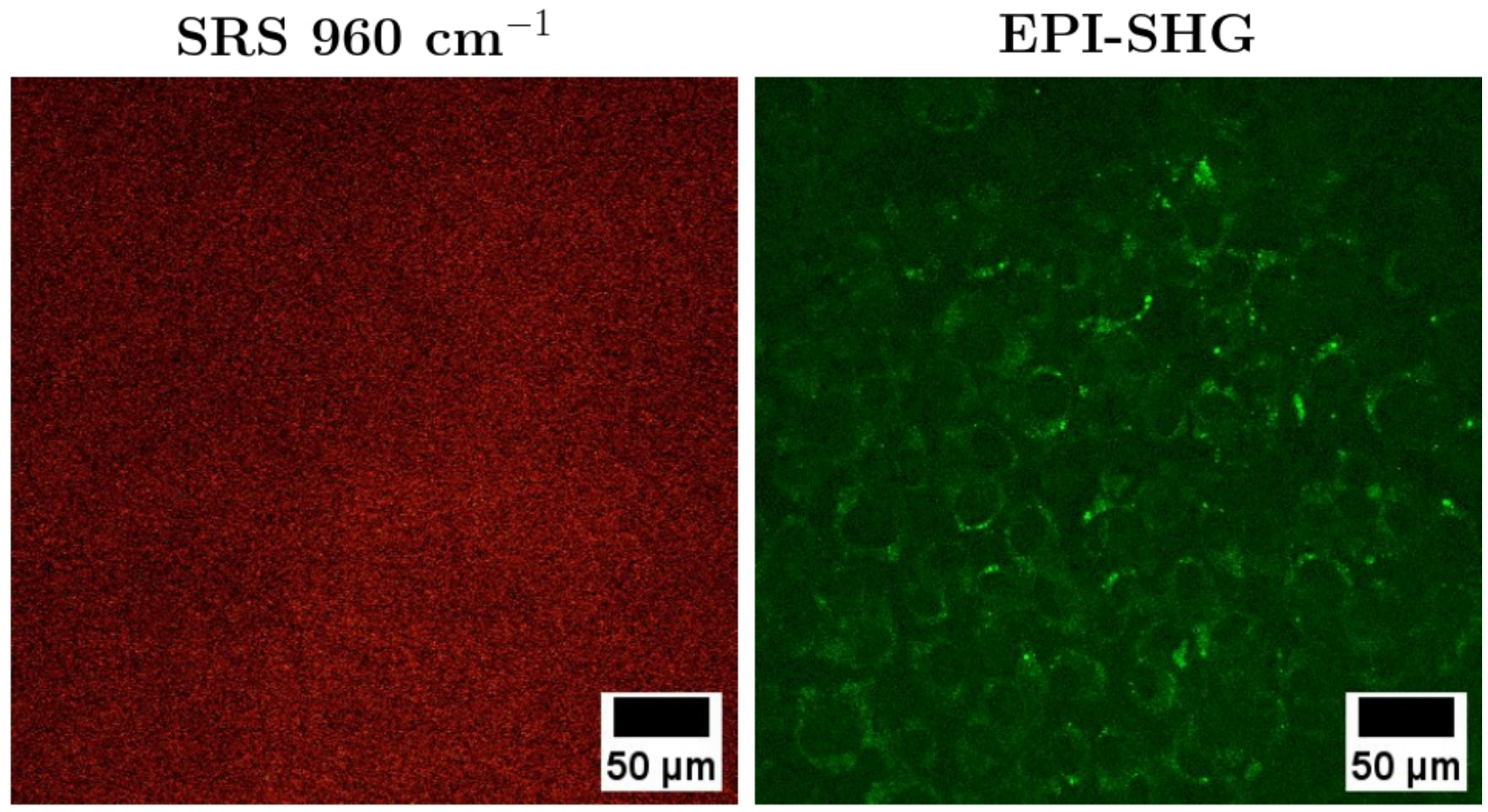

Figure S3: SRS and epi-SHG images of MC3T3-E1 monolayer cells cultured in RM for 4 weeks on polystyrene slides. On the left, SRS image at a Raman shift of 960 cm^{-1}. It can be observed that the SRS signal is dominated by the polystyrene slide background. On the right, epi-SHG image, where auto-fuorescence originating from the cells can be noticed.

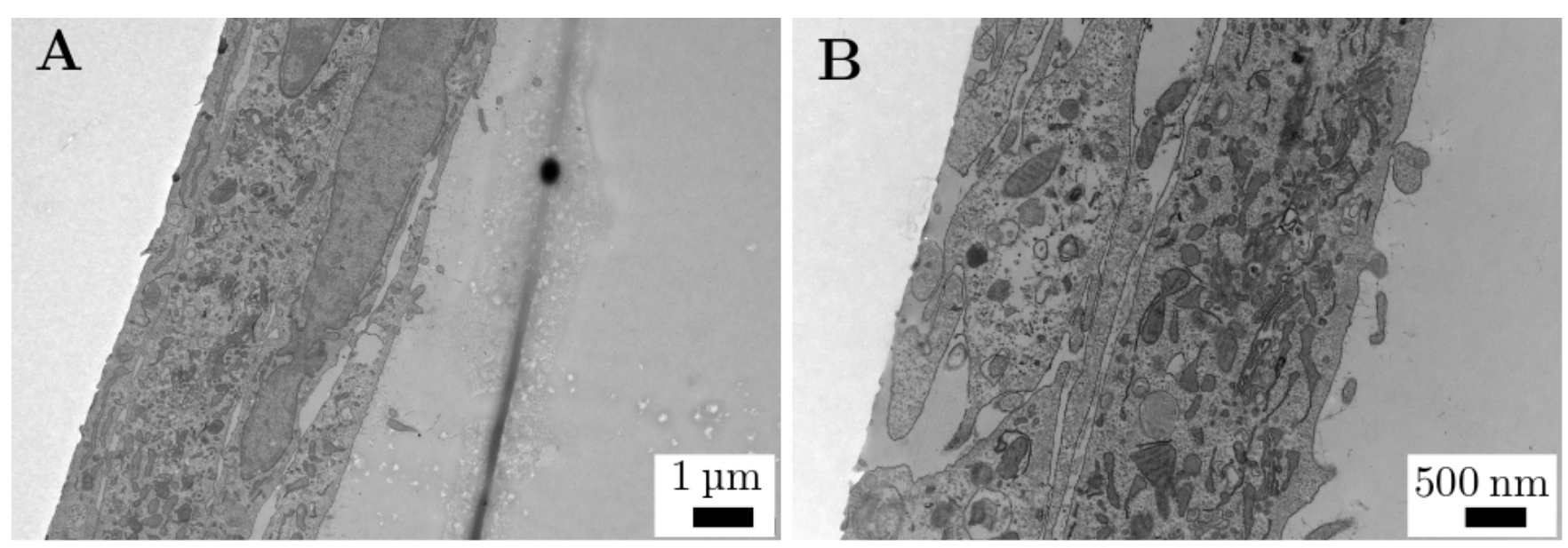

Figure S4: TEM images of MC3T3-E1 monolayer cells cultured in RM for 3 weeks. A) Low magnification image showing multiple cells, placed one on top of the other. It can be noticed the absence of any ECM. B) High magnification image showing multiple cells and the absence of any ECM.

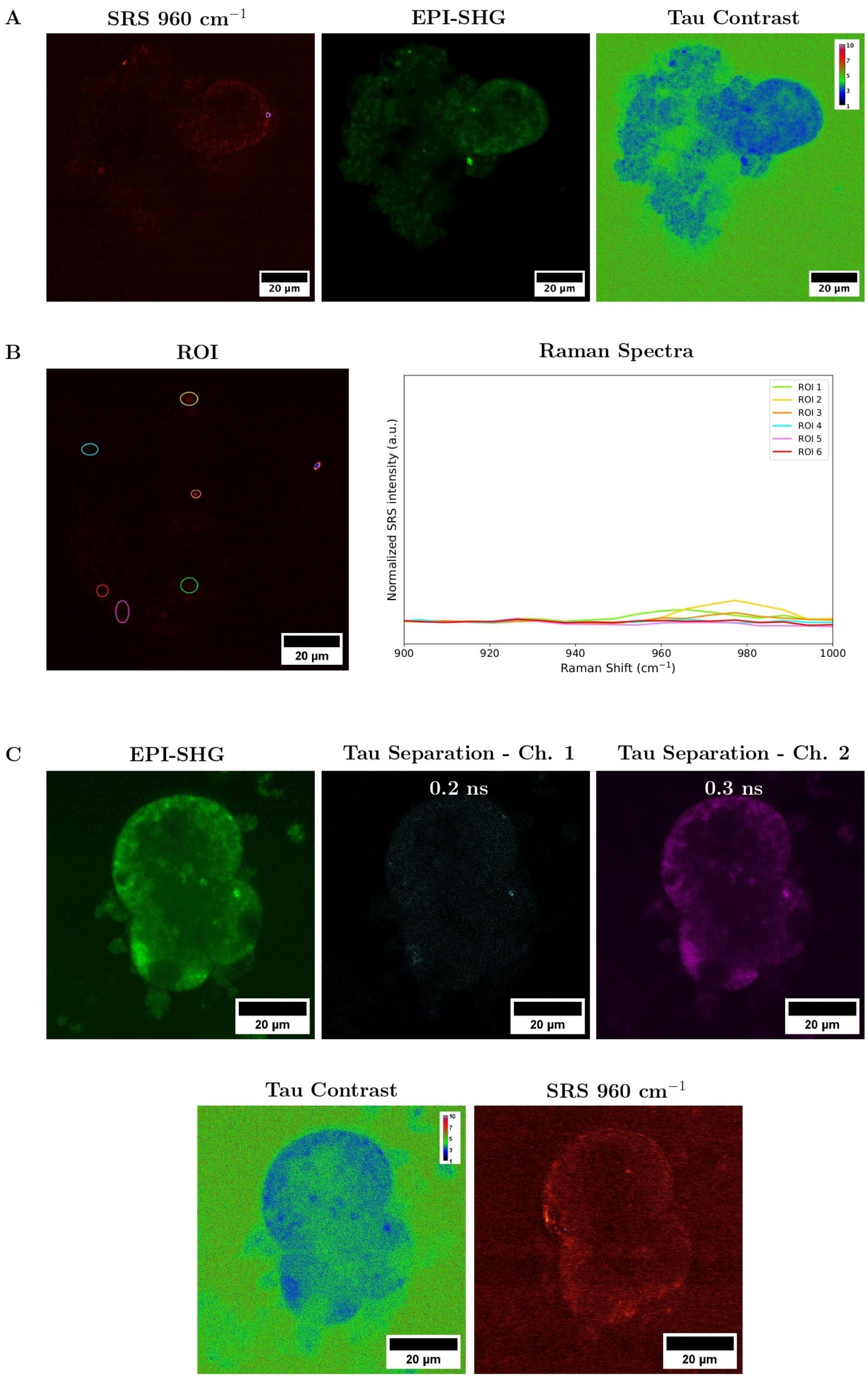

Figure S5: SRS and SHG analysis of mineralized ECM in alginate-encapsulated bone spheroids cultured for 4 weeks in RM. A) Combined SRS and epi-SHG images of a representative spheroid. The SRS image was obtained at Raman shift of 960 cm^{-1} to detect phosphate groups associated with mineralized deposits. It can be observed that no signal was detected, indicating the absence of phosphate groups. The epi-SHG signal consist of contributions from the collagenous matrix and cellular auto-fluorescence; the signals were distinguished using the TauContrast function. The calibration bar represents the average arrival time in nanoseconds. Absence of collagen signal can be observed. B) Raman spectra acquired in the 900 to 1000 cm^{-1} range and corresponding SRS image, indicating the selected regions of interest (ROIs). C) Epi-SHG images showing the signal contributions and the corresponding Tau-Separated channels, allowing the discrimination of collagen-derived SHG and auto-fluorescence based signal based on effective photon lifetime. No signal was detected for the fast channel associated with SHG signal, indicating the absence of deposited collagen. The TauContrast image and SRS image at 960 cm^{-1} were collected simultaneously. The calibration bar in the Tau Contrast image represents the average arrival time in nanoseconds.

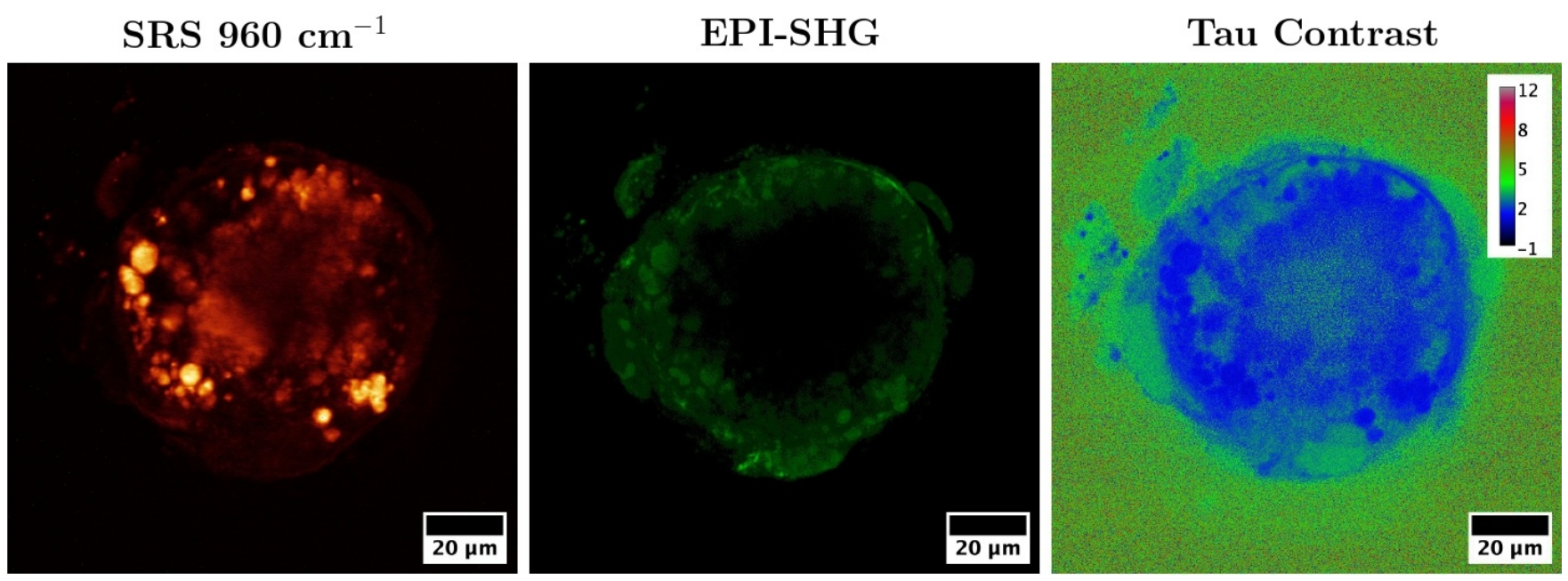

Figure S6: Validation of label-free SRS imaging through comparison with calcein fluorescence. Combined SRS, two-photon fluorescence and TauContrast images. Partial overlap was observed between SRS-positive regions in the outer layers of the spheroids and calcein-positive areas, indicating co-localization of phosphate-rich deposits with calcium-labeled structures. TauContrast was used to identify any change in the lifetime of the calcein-stained regions, compared to unstained samples.

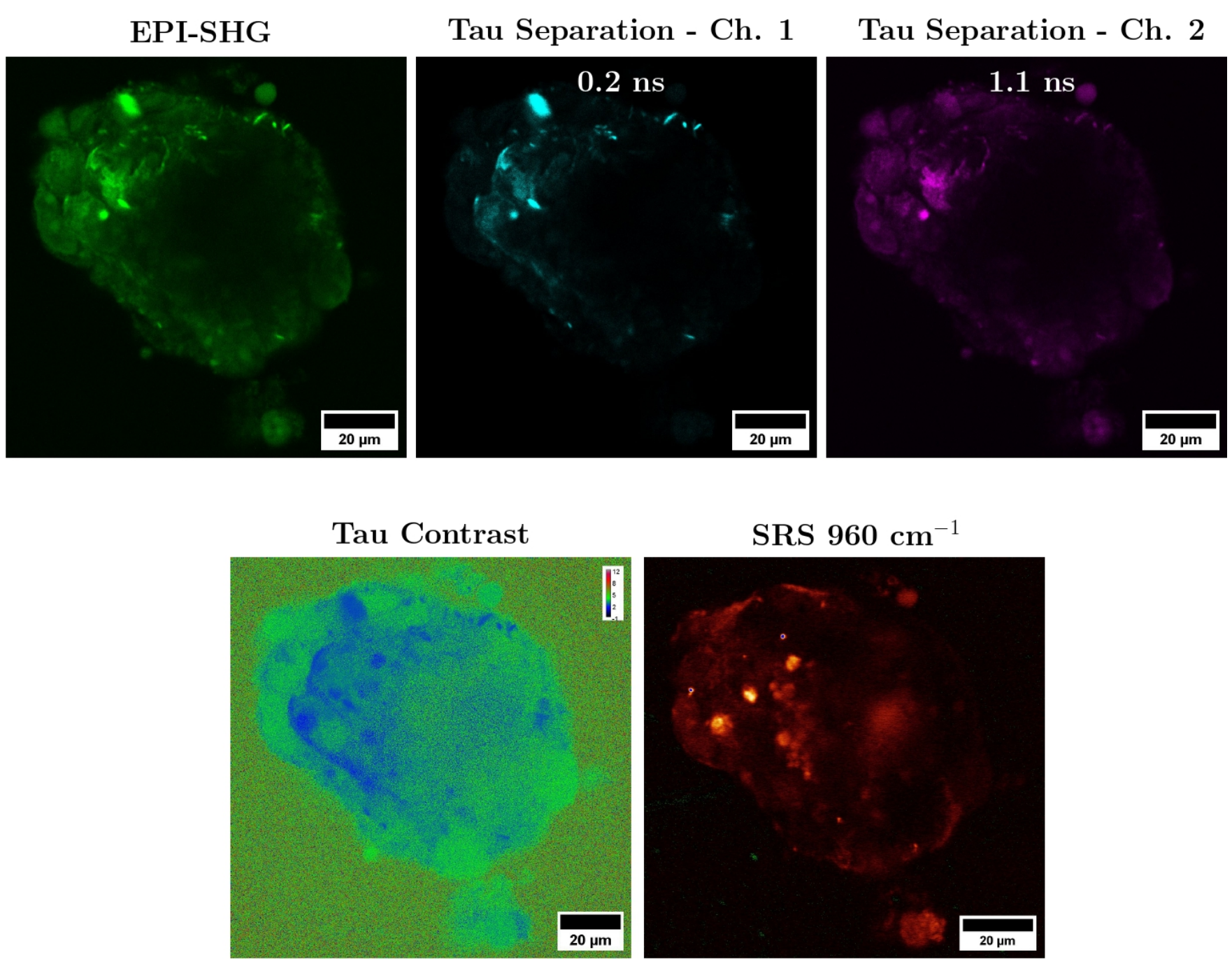

Figure S7: Epi-SHG image of a representative spheroid cultured in calcein-supplemented OM media for 4 weeks. Tau Separation functionality was activated to distinguish between the different signal contributions, allowing discrimination between SHG and auto-fluorescence and calcein signal. Tau contrast and SRS image at 960 cm^{-1} were also collected. The calibration bar in the tau Contrast image represent the average arrival-time in nanoseconds.
